# Recent advances in voltage-gated sodium channels, their pharmacology, and related diseases

**DOI:** 10.3389/fphar.2013.00052

**Published:** 2013-04-18

**Authors:** Jean-François Desaphy, Mohamed Chahine

**Affiliations:** ^1^Section of Pharmacology, Department of Pharmacy and Drug Sciences, University of Bari – Aldo MoroBari, Italy; ^2^Department of Medicine, Centre de Recherche de l'IUSMQ, Laval UniversityQuébec, QC, Canada

Because of their fundamental role in generating electrical impulses in many excitable tissues, sodium channels were among the first voltage-gated ion channels to be extensively investigated. Neurons bathed in a physiological solution containing 150 mM sodium ions respond to a threshold electrical stimulus by generating an action potential, whereas such a response is abolished in a Na^+^-free medium. Since the classic 1952 studies of squid axon sodium conductance, the Hodgkin and Huxley model of sodium channel gating has served as a framework for understanding the time and voltage-dependent properties of these channels (Hodgkin and Huxley, 1952). The advent of sophisticated biochemical and molecular approaches eventually lead to sodium channel purification (Hartshorne and Catterall, 1981) and cloning (Noda et al., 1984). To date nine genes encoding voltage-gated sodium channels are found in the human genome. Dysfunction of these channels causes diseases known as sodium channelopathies. In the 1990's, the term “channelopathy” was first coined to describe skeletal muscle hereditary diseases, including periodic paralysis and myotonia, due to mutations in the SCN4A gene encoding the muscle isoform of voltage-gated sodium channels (Wang et al., 1993). Many of these aspects are reviewed in this special issue dedicated to voltage-gated sodium channels (Simkin and Bendahhou, 2011; Savio-Galimberti et al., 2012).

Despite this impressive track record, there are still a number of critical questions that need to be addressed regarding voltage-gated sodium channels. Up to day, many studies have focused on the main sodium channel α-subunit because it contains all the requisites for a functioning channel, but it has become clear that the α-subunit interacts with auxiliary β-subunits and other protein partners, that regulate the trafficking, the expression levels and the function of these channels (Brackenbury and Isom, 2011; Chahine and O'Leary, 2011; Savio-Galimberti et al., 2012).

Today voltage-gated sodium channels are the primary targets of drugs used as local anaesthetics, antiarrhythmics, anti-convulsants, and neuroprotectants (Conte Camerino et al., 2007). Several ongoing studies are aimed at understanding the intimate drug-channel molecular interactions to design more efficacious and safer drugs (Fozzard et al., 2011; Desaphy et al., 2012; Morris et al., 2012). Gating properties of these channels are also affected during trauma injury (Morris et al., 2012). As far as our knowledge increases regarding sodium channel biophysics and involvement in diseases, sodium channels represent a more, and more attractive druggable targets for other conditions such as neuropathic pain and general anesthesia (Theile and Cummins, 2011; Herold and Hemmings, 2012). Voltage-gated sodium channels are also the targets of numerous natural ligands, especially neurotoxins, which provide important tools for the definition of the channel structure–activity relationship, and ideally may serve as lead compounds in the development of novel drugs (Stevens et al., 2011). Thus, voltage-gated sodium channels will likely continue to exert much interest for basic scientists and the pharmaceutical industry. This topic is dedicated to voltage-gated sodium channels their pharmacology and related diseases.
